# Systematic review of the venous thromboembolism risk assessment models used in aesthetic plastic surgery

**DOI:** 10.1016/j.jpra.2021.07.010

**Published:** 2021-08-11

**Authors:** Amelia J. White, Muholan Kanapathy, Dariush Nikkhah, Mo Akhavani

**Affiliations:** 1 Department of Plastic and Reconstructive Surgery, Royal Free NHS Foundation Trust Hospital, London, United Kingdom; 2 Division of Surgery & Interventional Science, University College London, London, United Kingdom

**Keywords:** Plastic surgery, venous thromboembolism, guidelines, risk assessment model, prevention, aesthetic surgery

## Abstract

**Background:**

A reliable venous thromboembolism (VTE) risk assessment model (RAM) can assist surgeons in identifying patients who would benefit from VTE prophylaxis. This systematic review was aimed at summarising the current available evidence on VTE RAMs used in aesthetic plastic surgery.

**Methods:**

A comprehensive search was performed in the PubMed, EMBASE and Cochrane databases to include primary studies describing VTE RAMs in aesthetic plastic surgery from 1946 to February 2019. The objective was to compare the different VTE RAMs described for aesthetic plastic surgery to recommend a reliable model to stratify patients.

**Results:**

Of the 557 articles identified in the PubMed, EMBASE and Cochrane databases, six articles were included in the final review. Five different RAMs were used in the included studies: Caprini 2005 RAM, Caprini 2010 RAM, Davison-Caprini 2004 RAM, the American Society of Anaesthesiologist's (ASA) physical status grading system and a tool developed by Wes et al. The difference in risk weightage amongst the tools along with the VTE incidences for different categories was compared. The Caprini 2005 RAM was the most widely reported tool and validated in plastic surgery patients.

**Conclusion:**

Amongst the five different tools currently used, the Caprini 2005 RAM was the most widely reported. This tool was validated in plastic surgery patients and reported to be a sensitive and reliable tool for VTE risk stratification; therefore, current data support its use until further higher quality evidence becomes available. Because of the heterogeneity of the data and low quality of the current evidence, a definitive recommendation cannot be made on the best VTE RAM for patients undergoing aesthetic plastic surgery. This paper highlights the need for randomised controlled trials evaluating the various RAMs which are essential to support future recommendations and guidelines.

## Introduction

Venous thromboembolism (VTE) is a major and largely preventable cause of morbidity and mortality following any surgical procedure. VTE affects approximately 1 in every 1000 of the UK population, which is the leading cause of preventable deaths in hospital, while the annual incidence of VTE in the American population is around 900,000, with a third of these patients reported to develop a fatal pulmonary embolism (PE)^1^^,^ ^2^. The rate of recurrence of VTE over 10 years is around 30%; hence, steps taken to reduce the incidence of VTE is of paramount importance.

The incidence of VTE in aesthetic surgery cases was found to be relatively low at 0.09%, however, this risk significantly increased with combined procedures^3^. The incidence of symptomatic VTE was reported to be high after post-bariatric body contouring surgery, especially when combined with circumferential abdominoplasty (7.7%), abdominoplasty (5.0%) and breast or upper body contouring (2.9%) procedures^4^. An overall rate of 1.1 % of PE was reported in abdominoplasty patients, mostly those with a combined intra-abdominal procedure^5^, while 23% of deaths following liposuction were attributable to PE^6^. Furthermore, VTE also has significant financial healthcare costs partly due to the high rates of recurrence and morbidity associated with the disease^7^.

VTE is a multifactorial disease. To evaluate VTE risk of patients pre-operatively, an individualised patient risk stratification tool is required. Failing to recognise VTE risk in predisposed patients will lead to underutilisation of prophylaxis measures; however, overtreating may also lead to its own complications. A good risk stratification tool would improve a surgeons’ ability to segregate patients based on their risk, provide information on the risk and benefits of treatment based on the risk level, and assist the surgeon in making a decision to proceed with a procedure^8^. Currently, several different risk stratification models exist, however, there are no systematic reviews comparing these models in patients undergoing aesthetic plastic surgery procedures. This leaves many plastic surgeons to use their discretion and personal experience to risk stratify patients rather than validated RAMs.

This systematic review aims to summarise the current available evidence on VTE assessment tools used in aesthetic plastic surgery. The various VTE risk stratification tools used in plastic surgery were evaluated to compare their reliability and outcome.

## Methods

The protocol for this systematic review was registered with PROSPERO international prospective registration of systematic reviews (registration number: CRD42019127297). A completed PRISMA checklist can be found in Appendix A.

### Search Strategies

A systematic search was performed from 1946 to February 2019 using the PubMed, EMBASE and Cochrane databases to identify studies of relevance to this review. The search strategy included a combination of text words and Medical Subject Headings (MeSH) terms. No language or publication restrictions were applied.

A sample search strategy for MEDLINE (OvidSP) is shown and a similar strategy was adapted for other databases:

1) ‘Plastic surgery’ AND [‘venous thromboembolism’ OR ‘thrombosis’] AND [‘prophylaxis’ OR ‘prevention’ OR ‘guideline’ OR ‘assessment]).

### Inclusion criteria

All primary studies describing VTE risk assessment and stratification tools in aesthetic plastic surgical patients were included.

### Exclusion criteria

The exclusion criteria were case reports and case series of less than ten patients, studies describing VTE assessment tools in non-plastic surgical procedures, studies describing VTE prophylaxis methods only, non-English language articles and studies in animal models.

### Objectives

The objective was to compare the different VTE risk assessment tools described in the literature for aesthetic plastic surgery in order to recommend surgeons with a reliable risk assessment model (RAM) to stratify patients.

### Study selection and data management

Study selection was conducted in a two-stage process. The titles and abstracts were initially screened by two reviewers (AW and MK) for potential eligibility, after excluding duplicate records. Next, studies identified as relevant underwent full-text review by both reviewers. Any discrepancies between the reviewers were resolved by discussion. The data from all full-text articles accepted for the final analysis were independently retrieved by AW and MK using a standardised data extraction form. Any discrepancies between the reviewers were resolved by discussion or referred to a third reviewer (MA). The search results, including abstracts, full-text articles and record of reviewer's decisions, including reasons for exclusion, were recorded in Endnote X8 (Clarivate Analytics, USA). Data were extracted from the studies as presented.

### Assessment of risk of bias of included studies

Risk of bias assessment was performed using the ROBINS-I (Risk Of Bias In Non-Randomized Studies – of Interventions) tool^9^.

#### Data analysis and synthesis

Narrative synthesis was performed to summarize the difference in the various risk assessment tools reported in the included articles and a narrative comparison was made on the difference in VTE incidence amongst the patients who were risk-stratified using the various tools.

## Results

### Literature search results

**Fig. 1:**
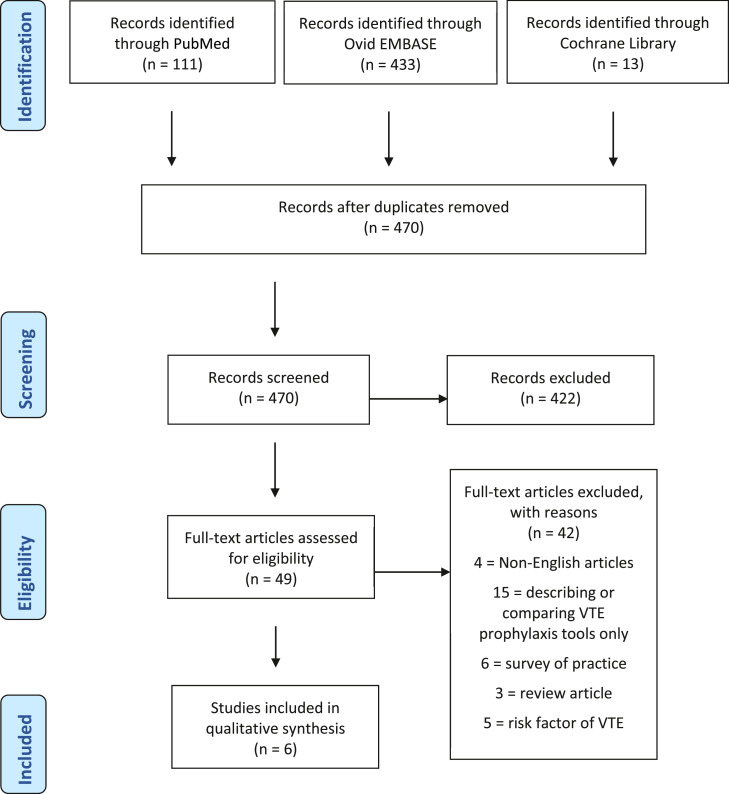
The PRISMA (Preferred Reporting Items for Systematic Reviews and Meta-Analyses) flow diagram.

A total of 557 articles were found from the PubMed, EMBASE and Cochrane Library database searches (Figure 1). After removing duplicates, 470 articles were screened. Of these, 421 articles did not meet the inclusion criteria and were therefore excluded. Full-text review was performed for the remaining 49 articles and, of these, 42 articles were excluded. A total of six articles were included in the systematic review^4^^,^ ^8^^,^ 10, 11, 12, 13. Cross-checking of the reference list revealed that no article was missed by the initial search. All the included papers were retrospective of cases or databases. There was no randomised controlled trial. Details of the included studies are summarised in Table 1. Risk of bias assessment is summarised in Table 2, whereby all included studies were at serious risk of bias.

**Table 1:** Overview of the included articles

Citation	Hatef et al. 2008^4^	Pannucci et al. 2011^10^	Pannucci et al. 2011^11^	Pannucci et al. 2012^8^	Shaikh et al. 2016^13^	Wes et al. 2015^12^
**Year**	2008	2011	2011	2012	2016	2015
**Country**	US	US	US	US	US	US
**Study design**	Retrospective review	Retrospective review	Retrospective review	Retrospective self-controlled study	Retrospective review	Review of national database
**Number of subjects**	347	1126	3334	3334	1598	17774
***Male***	n/a	n/a	1148	n/a	308	968
***Female***	n/a	n/a	2186	n/a	1290	16806
**Age (years)**	n/a	n/a	48..7 –50.3	n/a	49.7–51.8	45–65
**Co-morbidities**	Obesity, hormone therapy	n/a	High BMI	High BMI	Multiple	Multiple
**Type of surgery**	Abdominoplasty	Not specified	14 procedures	14 procedures	Not specified	Body contouring
**VTE Ram used**	Davison-Caprini 2004	Caprini 2005	Caprini 2005	Caprini 2005 vs Caprini 2010	Caprini 2005 vs ASA grading system	A tool developed by the team
**Incidence of VTE**	2.8%	1.69%	n/a	2.52%	1.5%	0.56%
**Adverse events**	Enoxaparin associated with higher bleeding rate	n/a	Nil	n/a	n/a	Nil
**Length of follow up**	n/a	60 days	60 days	60 days	30 days	30 days
**Statistical analysis model used**	Logistic regression	Kaplan–Meier	Multivariate logistic regression	Wilcoxon signed-rank test	Mann–Whitney U test	Multivariate logistic regression

**Table 2:** 

Citation	Pre-intervention		At Intervention	Post-intervention				Overall risk of bias judgement
	Bias due to confounding	Bias in selection of participants into the study	Bias in classification of interventions	Bias due to deviations from intended interventions	Bias due to missing data	Bias in measurement of the outcome	Bias in selection of the reported result	
Hatef et.al 2008^4^	Moderate risk	Moderate risk	Serious risk	Moderate risk	Moderate risk	Serious risk	Moderate risk	Serious risk
Pannucci et.al 2011^10^	Moderate risk	Moderate risk	Serious risk	Moderate risk	Low risk	Serious risk	Moderate risk	Serious risk
Pannucci et.al 2011^11^	Moderate risk	Moderate risk	Moderate risk	Low risk	Low risk	Serious risk	Moderate risk	Serious risk
Pannucci et.al 2012^8^	Moderate risk	Low risk	Low risk	Low risk	Low risk	Serious risk	Moderate risk	Serious risk
Wes et.al 2015^12^	Serious risk	Serious risk	Moderate risk	Serious risk	Serious risk	Moderate risk	Moderate risk	Serious risk
Shaikh et.al 2016^13^	No information	Moderate risk	Moderate risk	No information	Moderate risk	Serious risk	Moderate risk	Serious risk

### Description of the Risk Assessment Models

A total of five different tools were used in the included studies to predict risk of VTE: Caprini 2005 RAM, Caprini 2010 RAM, Davison-Caprini 2004 RAM, the American Society of Anaesthesiologist's (ASA) physical status grading system and a risk-scoring model developed by Wes et.al. A summary comparing the components of the different risk assessment tools along with the weightages of the risk factors is included in Table 3. In every RAM, each patient's total VTE risk score was calculated by adding the individual risk scores for all risk factors present. Patients were then categorised by the total score into different risk groups, ranging from the lowest to the highest risk. Table 4 summarises the different risk categories of all the RAMs used in the included articles.

**Table 3:** Comparing the components of the different risk assessment models based on weightage for risk factor

	Caprini 2005	Caprini 2010	Caprini-Davison	Tool by
	RAM	RAM	RAM (2004)	Wes et al.
**Age (in years)**				
40–59		1		
40–60			1	
41–60	1			
45–65				1
>60			2	
61–74	2	2		
>65				2
>75	3	3		
**Obesity**				
BMI >25	1			
BMI >30		1		
BMI 30–34.9				2
BMI >35				3
BMI >40		2		
BMI >50		3		
>20% IBW			1	
**Operative details**				
**Trunk contouring**				2
>2 regions contoured				2
Minor surgery	1	1	1	
Prior major surgery (<1 month)	1	1		
Major surgery (>45 mins)	2		2	
Major surgery (<60 mins)		2		
Major surgery (2–3 h)		3		
Major surgery (>3 h)		5		
Free flap			3	
Arthroscopic surgery	2			
Arthroscopic surgery (>60 mins)		2		
Laparoscopic surgery (>45 mins)	2			
Laparoscopic surgery (>60 mins)		2		
Major lower extremity arthroplasty	5	5		
**Patient factors**				
History of DVT/PE	3	3	3	
Malignancy (previous or current)	2	2	2	
Present chemotherapy		3		
Congestive heart failure	1	1	3	
Acute MI	1	1		
Previous MI			3	
CVA/TIA	5	5	5	
Genetic hypercoagulable disorder	3	3	3	
Acquired hypercoagulable disorder	3	3		
Varicose veins	1	1		
History of IBD	1	1		
Swollen legs	1	1		
Sepsis (<1 month)	1	1	3	
Serious lung disease	1	1		
COPD	1	1		
Family history of thrombosis	3	3		
Multiple trauma (<1 month)	5	5	5	
Acute spinal cord injury (<1 month)	5	5	5	
History of SVT		3		
Blood transfusion (<1 month)		1		
Central venous access	2	1	2	
Hip/pelvis/leg fracture	5	5	5	
**Female patients**				
Pregnancy or <1month postpartum	1	1	1	
Oral contraceptive/HRT	1	1	1	
History of unexplained stillbirth	1	1		
Recurrent spontaneous abortion	1	1		
Premature birth	1	1		
Immobilisation				
**Inpatient**				2
Confined to bed for >72 h	2		2	
Medical patient at bed rest	1	1		
Leg plaster cast/brace	2	1	2	
**Others**				
Wound class: non-clean				1

Each number denotes weightage for each risk factor. The overall score for each patient is then used to assign patients to different risk categories.

*Legend: IBW – ideal body weight, MI – myocardial infarction, CVA – cerebral vascular accident, TIA – transient ischaemic attack, IBD – inflammatory bowel disease, COPD – chronic obstructive pulmonary disease, SVT – superficial venous thrombophlebitis, HRT – hormone replacement therapy*

**Table 4:** Definition of risk groups for the different RAMs. Each RAM assigned patients into different risk groups based on the number of risk factors that they score.

Definition of risk groups	Lowest risk groups	Low risk groups	Moderate risk groups	High risk groups	Highest risk groups
**Caprini 2005 RAM**	1*–*2 factors	3*–*4 factors	5*–*6 factors	7*–*8 factors	>8 factors
**Caprini 2010 RAM**	1*–*2 factors	3*–*4 factors	5*–*6 factors	7*–*8 factors	>8 factors
**ASA PA grading system**	*–*	ASA score 1*–*2	-	ASA score 3+	-
**Davison-Caprini RAM (2004)**	*–*	1 factor	2 factors	3*–*4 factors	>4 factors
**Tool by Wes et al**	*–*	0*–*4 factors	5*–*7 factors	8*–*10 factors	-

The Caprini 2005 RAM was the most widely used tool whereby it was reported in four of the included articles. The Caprini 2005 RAM, which was a modification of that initially published in 1991^14^, was a weighted risk stratification tool which produces an aggregate risk score based on the presence or absence of 39 individual risk factors. Based on these scores, patients were then categorised into five groups: lowest risk, low risk, moderate risk, high risk and super high risk. The Caprini 2010 RAM, which was a modification of the 2005 RAM, had four distinct changes, including addition of new risk factors or re-weighting of old risk factors. This tool utilised additional sub-categorization for body mass index (BMI), operative time and cancer risk factors^8^.

On the other hand, the Davison-Caprini 2004 RAM was a modification of the Caprini model to make it more relevant for plastic surgery patients^15^. It was simplified and included additional risk factors specific to plastic surgery, such as free flap, while unrelated risks, such as arthroscopic or laparoscopic surgery, were removed. This tool divided patients into four risk groups: low, moderate, high and highest.

Similarly, Wes et al. developed a new tool including just eight risk factors using the American College of Surgeons National Surgical Quality Improvement Program datasets to identify predictors of VTE in patients undergoing body contouring procedures. A multivariate logistic regression was performed on the dataset to identify independent predictors of VTE. Based on the risk factor scores, patients were assigned to three risk groups: low, medium and high. The use of this tool was only reported in one of the included articles^12^.

In contrast, the ASA grading system, which was initially developed to classify patients pre-operatively based on their co-morbidities, was adopted to risk stratify VTE risk as patients with high pre-operative risk score were noted to have a greater risk of peri-operative complications including VTE. For VTE risk stratification, patients were divided into two groups: low-risk group (scores 1–2) and high-risk group (scores 3–5). This tool was used in one article which compared its outcome with Caprini 2005 RAM^13^.

In general, all five different reported tools included patient demographics, such as age, BMI or obesity, previous history or VTE and the use of hormone replacement therapy or oral contraceptive pills as risk factors. However, the tool developed by Wes et al. did not include previous history of VTE or the use of hormone therapy and oral contraceptive pills as a risk factor. The Caprini RAMs were most comprehensive, taking into consideration the greatest number of different risk factors for stratifying patients, while the ASA tool was the least specific as individual risk factors were not scored separately, instead risk factors were grouped together in each category.

The tool developed by Wes et al. and the Davison-Caprini RAM were specifically developed, and evaluated in body contouring patients. The other tools were developed for general surgical patients who were adopted to risk stratify plastic surgery patients.

### Comparison of VTE incidence related to the Risk Assessment Models

The incidences of VTE based on the different risk categories for each RAM are summarised in Table 5. Generally, similar trend of increasing rates of VTE with worsening risk category was seen across the different RAMs. However, Caprini 2010 RAM demonstrated a decreasing rate of VTE with worsening risk, whereby the rate of VTE in the “lowest” risk group (0.72%) was higher compared with the “low”-risk group (0.43%). The rate of VTE in the “low”-risk group was, however, comparable with that of the Caprini 2005 RAM (0.43% vs 0.42%). The Caprini 2010 RAM was directly compared against Caprini 2005 RAM in one of the studies which retrospectively risk-stratified 3334 patients using both the RAMs and compared the VTE incidence^8^. The authors reported that the 2005 model separated the high-risk from low-risk patients better than the 2010 model. Patients classified as “super-high” risk using the Caprini 2005 RAM were significantly more likely to have had a 60-day VTE event when compared with patients classified as “super-high” risk using the 2010 model, with no significant differences observed in 60-day VTE rate at any other distinct risk levels. Based on these data, the authors concluded that the Caprini 2005 RAM is a better predictor of VTE risk for plastic surgery patients.

**Table 5:** VTE incidence reported with each of the different RAMs

Rate of VTE %	Lowest risk	Low risk	Moderate risk	High risk	Super high risk
**Caprini 2005 RAM****^8^**	0.00	0.42	1.21	1.87	5.85
**Caprini 2010 RAM****^8^**	0.72	0.43	1.17	1.20	2.52
**ASA PA grading system****^13^**	n/a	0.88	n/a	3.71	n/a
**Davison-Caprini RAM (2004)****^4^**	n/a	*–*	*–*	0.57	4.89
**Tool by Wes et.al****^12^**	*–*	0.14	0.97	2.95	*–*

The Caprini 2005 RAM was also compared against the ASA grading system, in a study which retrospectively risk-stratified 1598 patients using both tools and compared the incidence of VTE^13^. The authors reported that the Caprini 2005 RAM concentrated a higher percentage of patients with VTE in the high-risk category as compared with the ASA grading system, directly corresponding to the increase in patient's odds of DVT and PE. The sensitivity of the Caprini model was noted to be higher 0.708 (confidence interval: 0.489–0.874)) compared with the ASA model (confidence interval: 0.542 (0.328–0.745)). The authors further mentioned that the sensitivity and specificity for VTE incidence increased when the ASA and Caprini models were used in combination, therefore recommended combining the two grading systems. However, this was the only study that used the ASA grading system to evaluate VTE risk.

The other two studies that solely evaluated Caprini 2005 RAM reported that the tool effectively stratified patients for VTE risk, with patients with a higher score having a greater likelihood of VTE events, while notable risk reduction was present in patients with a higher score who received post-operative enoxaparin^10^^,^ ^11^.

Only one study evaluated the use of Davison-Caprini RAM^4^. The authors reported that this model is a useful tool for assigning VTE risk as 89.5% of patients who developed VTE were stratified in the highest risk group. Similarly, Wes et al., who proposed a new RAM, reported that the patients stratified into the high-risk group experienced a 21-fold increase in VTE incidence compared with the low-risk cohort. However, these two tools were not compared against other available tools to demonstrate the difference in the outcome of VTE incidence.

#### VTE prophylaxis regimes

Post-operative chemoprophylaxis was a focus of one study that reviewed 347 patients who underwent excisional body contouring procedures between 2003 and 2006^4^. Patients were risk-stratified using the Davison-Caprini RAM. Patients were divided into two groups; one group (comprising 137 patients) were treated with enoxaparin, sequential compression devices and early ambulation and the second group (comprising 221 patients) were treated with just sequential compression devices and early ambulation. In the group prescribed enoxaparin, 49 patients received their first dose of enoxaparin <2 hours pre-operatively, while 88 patients received their first dose either intra-operatively or <2 hours post-operatively. The incidence of VTE was unchanged in all patients by the administration of enoxaparin. However, enoxaparin did lead to a statistically significant decrease in DVT in patients undergoing circumferential abdominoplasty (p=0.0064). The administration of enoxaparin also lead to a significant increase in haematoma formation (p=<0.001). The timing of administration of enoxaparin was not clinically significant for VTE incidence.

This underscores the difficulty encountered with the dependence on RAMs for the use of chemoprophylaxis.

## Discussion

The aim of this article was to summarise the current evidence on the different VTE assessment tools used in aesthetic plastic surgery and recommend a reliable risk stratification model for patients undergoing aesthetic plastic surgery. A good risk stratification tool would improve a clinician's ability to separate high-risk from low-risk patients and provide information on the risk and benefit of a treatment based on risk level and assist surgeons in making a decision for the treatment^8^.

We found six articles, and no randomised controlled trials currently exist. The current evidence involves retrospective case series and review of databases with huge heterogeneity in the study population and serious risk of bias. There were five different tools used to predict risk of VTE in patients undergoing aesthetic plastic surgery, with Caprini 2005 RAM being the most commonly used. The difference in the components of the different risk assessment tools along with the weightages of the risk factors was compared. The incidences of VTE based on the different risk categories for each RAM generally followed a similar trend of increasing rates of VTE with worsening risk category, however, demonstrated different sensitivity across the various tools. The difference in the predictability of VTE incidence by the various tools were not compared in a same population by any of the studies, hence, a recommendation on the most reliable tool to be used in aesthetic plastic surgery cannot be made. However, the Caprini 2005 RAM was compared against Caprini 2010 RAM and ASA grading system in two separate studies and the authors concluded that the Caprini 2005 RAM was a more reliable and sensitive tool. Similarly, two other studies that performed retrospective validation of the Caprini 2005 RAM reported it as an effective risk-stratification tool for VTE risk in plastic surgery.

The American Society of Plastic Surgeons (ASPS) previously published a VTE Task Force Report in 2012 that concluded that there was not enough evidence to make all-inclusive recommendation for plastic surgery prophylaxis regime^16^. However, the Caprini 2005 RAM was used as the reference point for their recommendations as it was formally validated to stratify plastic surgery patients^8^^,^ ^16^. The Caprini 2010 RAM was not selected because of the additional points allocated for longer surgery time, which is common in many plastic surgery procedures, and the inaccurate weight of points given to obesity which may lead to over scoring and artificially placing patients in a higher risk category. They also recommended that patients undergoing procedures, such as body contouring and abdominoplasty, should be considered for post-operative chemoprophylaxis agents^16^.

The type and duration of chemoprophylaxis was the focus of one study that compared the use of enoxaparin vs no enoxaparin and also the timings of administration of enoxaparin. They found no significant difference in the rates of VTE events between the groups, but there was a significant increase in haematoma formation in the enoxaparin group^4^. This emphasises the need for appropriate risk stratification of plastic surgery patients and further higher quality studies comparing different chemoprophylaxis regimes.

In a recent survey conducted by the American Society for Aesthetic Plastic Surgery (ASAPS) involving 286 responses, it was reported that 93.6% respondents utilise a risk assessment tool, of which the Caprini 2005 RAM being the most commonly used (74.2%), followed by the ASPS VTE Task Force recommendations (36.9%) and physician experience or preference (27.5%)^17^. Furthermore, several respondents reported that they were reluctant to use the Caprini RAM in their aesthetic practice due to overemphasis on chemoprophylaxis, inaccurate weightage given to patients with high BMI, inability to appropriately calculate risk of patients undergoing procedure under sedation or regional anaesthesia and lack of gradation of operative time. It should also be noted that the Caprini 2005 RAM consists of a 39-item checklist which could prove cumbersome for surgeons to use. These variations in practice and limitations of the RAM suggests the need of a streamlined, more practical guideline for aesthetic procedures supported by high-quality evidence, hence, the need for prospective trials evaluating the various RAMs.

There are several limitations in this study which include the following: the lack of high-quality level 1 evidence and the high risk of bias amongst the identified studies. Furthermore, the study design and study population of the included articles were heterogeneous which limited our ability to directly compare the reliability of the different tools and to recommend the best tool for future practice.

## Conclusion

This study outlined the various RAMs used in aesthetic plastic surgery and compared the VTE incidence amongst the different tools. Because of the heterogeneity of the data and low quality of the current evidence, a definitive recommendation cannot be made on the best VTE RAM for patients undergoing aesthetic plastic surgery. However, amongst the five different tools currently used to predict risk of VTE in patients undergoing aesthetic plastic surgery, the Caprini 2005 RAM was the most widely reported tool. This tool was validated in plastic surgery patients and was reported to be a sensitive and reliable tool for VTE risk stratification amongst aesthetic plastic surgery patients, therefore, we would support its use until further higher quality evidence becomes available. With regards to chemoprophylaxis regimes, there is currently not enough evidence to make an all-inclusive recommendation and further research in this area is crucial. As sufficient evidence is lacking on the best RAMs and chemoprophylaxis regimes to use, we would recommend that aesthetic plastic surgeons avoid surgery on patients who are at high risk of mortality from VTE using the Caprini 2005 RAM to identify this group of patients. This paper highlights the need for randomised controlled trials evaluating the various RAMs which are essential to support future recommendations and guidelines.

## Appendix Supplementary materials

Image, application 1
